# Capsule endoscopy retention in the upper esophagus: A comprehensive literature review

**DOI:** 10.1097/MD.0000000000035113

**Published:** 2023-09-08

**Authors:** Arteen Arzivian, Elke Wiseman, Yanna Ko

**Affiliations:** a Endoscopy Unit, Macquarie University Hospital, Macquarie Park, NSW.

**Keywords:** capsule endoscopy, cricopharyngeus, esophagus, laryngoscope, overtube, patency capsule, Zenker diverticulum

## Abstract

Capsule endoscopy is the first-line investigation for small bowel disorders. Capsule retention in the small bowel is the most common adverse event. Retention has also been reported in the upper esophagus; however, guidance for diagnosis and management is lacking. This review aims to summarize the diagnostic workup and management of this complication. We conducted a systematic literature review by searching 5 databases; relevant keywords and MeSH terms were used. Exclusion criteria included publications of non-adult patients in non-English languages. Data from eligible studies were analyzed using IBM SPSS 29. Twelve case reports were found (9 males, median age of 76 years); 10 capsule retentions in Zenker’s diverticulum and 2 in the cricopharyngeus. Most patients were asymptomatic before capsule endoscopy. Capsule retention was symptomatic in half of the patients (6/12). A neck X-ray confirmed the diagnosis in all patients. Endoscopic capsule retrieval was achieved by different tools (9/12) (Roth’s net was the most used tool, 6 patients); retrieval required rigid endoscopy in a few cases (3/12). Endoscopic capsule re-insertion was successful; using an overtube to bypass the upper esophagus was the safest method. In conclusion, capsule retention in the upper esophagus is uncommon yet exposes patients to the risk of unnecessary procedures. Symptoms of swallowing and medium-to-large size Zenker’s diverticulum should be considered contra-indications for capsule endoscopy. Neck and chest X-rays are required for elderly patients who do not pass the capsule 2 weeks after ingestion. Endoscopic retrieval using Roth’s net and re-insertion through an overtube should be considered first-line management.

## 1. Introduction

Capsule endoscopy (CE) was introduced to clinical practice over 2 decades ago.^[1]^ Since then, it has evolved significantly, and Its role has expanded to be considered the first-line technique of small bowel endoluminal imaging for multiple indications, including obscure gastrointestinal bleeding, Crohn’s disease, and Iron deficiency anemia.^[2–4]^ The yield of CE varies depending on the clinical indication; it has been reported as high as 73%, 71%, and 45% for “obscure gastrointestinal bleeding,” “suspected small bowel Crohn’s disease,” and “Iron deficiency anemia without overt gastrointestinal bleeding,” respectively. Less common indications include refractory or obscure Celiac disease, small bowel tumors and other uncommon conditions.^[5]^

The widespread use of CE is supported by its excellent safety profile in various age groups and clinical scenarios. Since its first use, the rate of adverse events has declined over the last 2 decades, especially with the ongoing technological development. Capsule retention (CR), aspiration, technical failure and incomplete examination are the most reported adverse events. The two most common risk factors for adverse events are Inflammatory Bowel Disease and old age. In a meta-analysis of adverse events over 2 decades,^[6]^ the rate of individual adverse events was 0% to 1%, and the rate of incomplete examination was 9% to 19%. These results are lower than the previously reported rates, which is reassuring for patients undergoing this procedure.

CR is the main adverse event of CE. It is defined as “The capsule remaining in the gastrointestinal tract for more than 2 weeks after ingestion.” The rate of occurrence of CR is reported to be around 1% to 2%^[7]^ and as high as 4% to 8% for Inflammatory Bowel Disease patients,^[8]^ with lower rates and numbers reported in a meta-analysis of these individual studies.^[6]^ Most retentions happen in the small bowel secondary to inflammatory or fibrotic strictures, less commonly due to tumors or previous surgeries and adhesions; this has been reviewed in multiple studies.^[9,10]^ A patency capsule can be used in these high-risk patients; it is made of lactose and coated with barium, which makes it dissolvable and radio-opaque; if the patency capsule is retained, then it will spontaneously biodegrade and dissolve with no complications and will indicate the unsafety to proceed with CE.

Capsule aspiration into the respiratory tract occurs in older adults with multiple co-morbidities; it is luckily a rare occurrence, with no more than 50 case reports in the literature over the last 2 decades. Despite being a horrific experience for patients, it is generally safely managed with 1 death report only; this was attributed to the multiple co-morbidities of the patient rather than a direct adverse outcome of capsule endoscopy.^[11]^

A less common but important complication is CR in the esophagus, mainly the upper esophagus. Contraindications to CE, according to the manufacturers, include “Dysphagia,” “Motility disorders,” and “Swallowing disorders.” This is similar to the contra-indications in both Canadian and American guidelines.^[3,4]^ These resources have not explicitly mentioned that CE is contraindicated in asymptomatic esophageal pathologies (e.g., Zenker’s diverticulum), although this has been considered in individual studies in the early 2000s.^[12]^ There is also a lack of guidance regarding the workup and management of patients with this complication. Hence, we conducted a comprehensive literature review to search for articles and reports on “Capsule endoscopy retention in the upper esophagus” to study and document the epidemiology, risk factors, methods of capsule retrieval and other practical details. Due to its rare occurrence and the lack of other evidence-based studies, this review can potentially work as a guide for gastroenterologists facing this clinical scenario.

## 2. Methods

We conducted an advanced search of studies and reports in 5 major databases, including “Medline,” “PubMed Central,” “Science Direct,” “Embase,” and “Scopus.” The keywords included [“Capsule Endoscopy retention” OR “Video capsule endoscopy retention” OR “Pillcam retention” OR “Wireless Endoscopy retention”] AND [“Esophagus” OR “Zenker’s Diverticulum” OR “Pharyngeal pouch” OR “Cricopharyngeus”]. The MeSH database was searched for MeSH terms related to the keywords and included in the search protocol appropriately. Exclusion criteria included publications for non-adult patients (below the age of 18 years) and publications in non-English languages.

Duplicates were removed using “Endnote 20” software. Records were screened, followed by reports screening for eligibility to be involved in the study. References cited in the reports on the same or similar topic were considered and examined for eligibility to be included in this review.

Data were extracted from each case report; the specific details gathered included the year of publication, the age of the patient, indication for capsule endoscopy, any risk factors for capsule retention in the esophagus, symptoms before or after capsule retention, time to diagnosis between capsule endoscopy ingestion and diagnosing retention, modalities and tests used for diagnosis, capsule retrieval modality and tools used for it, and endoscopic re-insertion method or device. IBM SPSS version 29.0 (Armonk, New York, USA) was used to conduct statistical analysis and compare findings among all case reports.

The X-ray and endoscopic images in this review were included after obtaining Informed consent from the patient and granting permission to publish photographs or other material that might identify them. The CR in this patient was not included in the data analysis.

## 3. Discussions/observations

Twelve case reports of CR in the upper esophagus were identified.^[13–24]^ Interestingly, one of the reports was for a patient with 2 capsules retained at the same time in a Zenker’s diverticulum; the failure of the first CE was interpreted as a system malfunction, an unremarkable plain abdominal radiograph 6 days after ingestion of the first CE provided a false reassurance that it has already passed, a second CE was ingested under Wi-Fi real-time monitoring confirming the failure and retention at the upper esophagus, a plain neck x-ray confirmed 2 foreign bodies in the neck consistent with 2 CEs.^[23]^ (Table 1) summarizes the case reports and the relevant extracted data.

**Table 1:** Eligible case reports and the relevant extracted data.

Study	Year	Age	Sex	Indication	Risk factor	Symptoms post CR	Time to diagnosis	Diagnosis	Location of CR	Retrieval	Re-insertion	Adverse outcomes
Fleischer et al^[13]^	2003	76	M	Melena	Dysphagia with large tablets	Foreign body	Not delayed	Clinical	Crico-pharyngeus	Endoscopy, Roth’s net	Dilatation & re-ingestion	No
Ford et al^[14]^	2005	73	F	Melena	Asymptomatic Zenker’s diverticulum	No	Not delayed	CE images, Neck-XR	Zenker’s diverticulum	Endoscopy	Not attempted	No
Knapp et al^[15]^	2005	79	M	IDA	Asymptomatic Zenker’s diverticulum	No	Not delayed	Neck-XR	Large Zenker’s diverticulum	Endoscopy, polypectomy snare	Not attempted	No
Simmons et al^[16]^	2005	73	M	Melena	Symptomatic Zenker’s diverticulum	Foreign body	Not delayed	Neck-XR	3 cm Zenker’s diverticulum	Endoscopy, polypectomy snare	Endoscopy, overtube, Savary dilator	No
Garza et al^[17]^	2006	83	M	IDA	Prior Difficult Esophageal Intubation	No	Not delayed	CE images, Neck-XR	Zenker’s diverticulum	Endoscopy, ERCP balloon catheter	Endoscopy, overtube, Roth’s net	No
Venecourt-Jackson et al^[18]^	2009	72	F	Melena	Symptomatic Zenker’s diverticulum	No	Not delayed	CE images, chest XR	Zenker’s diverticulum	Rigid Laryngo-scope	Patient declined	No
Nakaji et al^[19]^	2010	78	M	IDA	Previous Radiotherapy	Foreign body	Not delayed	Neck-XR	Crico-pharyngeus	Rigid laryngoscopy	Endoscopy , retrieval net	No
Horiuchi et al^[20]^	2011	66	M	Melena	None	Discomfort	Not delayed	CE images	Zenker’s diverticulum	Endoscopy, Roth’s net	Endoscopy	No
Ziachehabi et al^[21]^	2011	71	M	Melena	None	No	Not delayed	CE images, Neck-XR	9 cm Zenker’s diverticulum	Endoscopy, Roth’s net	Endoscopy	No
Kropf et al^[22]^	2013	82	F	Anaemia	None	Odyno-phagia	4 mo	Neck-XR	Zenker’s diverticulum	Rigid Laryngoscopy	Not attempted	Inflamed mucosa
Rondonotti et al^[23]^	2017	79	M	Melena	None	No	2 CEs	CE images, Neck-XR	5 cm Zenker’s diverticulum	Endoscopy, Roth’s net	Endoscopy, overtube	No
Rubiano et al^[24]^	2022	75	M	Melena	None	Dysphagia	2 yr	CT chest	Zenker’s diverticulum	Endoscopy, Roth’s net	Not attempted	No

CE = capsule endoscopy, CRM = capsule retention, IDA = iron deficiency anaemia.

CR in the upper esophagus was exclusively reported in elderly patients with a median age of 75.8 years, minimum age of 66 years, and maximum age of 83 years. The male-to-female ratio was 3:1. Both these findings correspond to the prevalence of Zenker’s diverticulum in the general population, which is reported to be more prevalent in men and between the seventh and eighth decade of life; the higher prevalence of Zenker’s diverticulum in these patients is likely related to the fibrosis and muscle necrosis of the upper esophageal sphincter predisposing to mucosal outpouching.^[25]^

The indication for CE in all reported cases was related to gastrointestinal bleeding; overt bleeding with melena and occult bleeding with iron deficiency anemia accounted for 76.9% and 23.1% of the cases, respectively. We believe that CR in the upper esophagus was not reported in patients with Crohn’s disease due to the younger age of these patients with lower prevalence of upper esophageal anatomical pathologies, and possibly due to more rigorous questioning and cautious approach when they undergo CE due to their higher risk of CR in the small bowel.

Pre-procedurally, nearly half (53.8%) of the patients had no CR risk factor identified on questioning. About one-third of them (4 patients, 30.8%) were known to have Zenker’s diverticulum (2 symptomatic and 2 asymptomatic), 1 patient (7.7%) had previously difficult esophageal intubation during transesophageal echocardiography leading to minor bleeding which retrospectively was thought to be related to undiagnosed Zenker’s diverticulum, and another patient (7.7%) had previous radiotherapy for laryngeal cancer 20 years prior CE procedure with no residual symptoms of swallowing; this predisposed him for CR in the cricopharyngeus. Regarding swallowing symptoms related to the upper esophagus, most patients (76.9%) were completely asymptomatic, while 23.1% reported having dysphagia before the procedure. The higher incidence of CR in asymptomatic patients indicates that despite considering “Dysphagia” and other swallowing symptoms as relative contra-indications for CE, it will not entirely prevent its occurrence.

**Figure 1.**
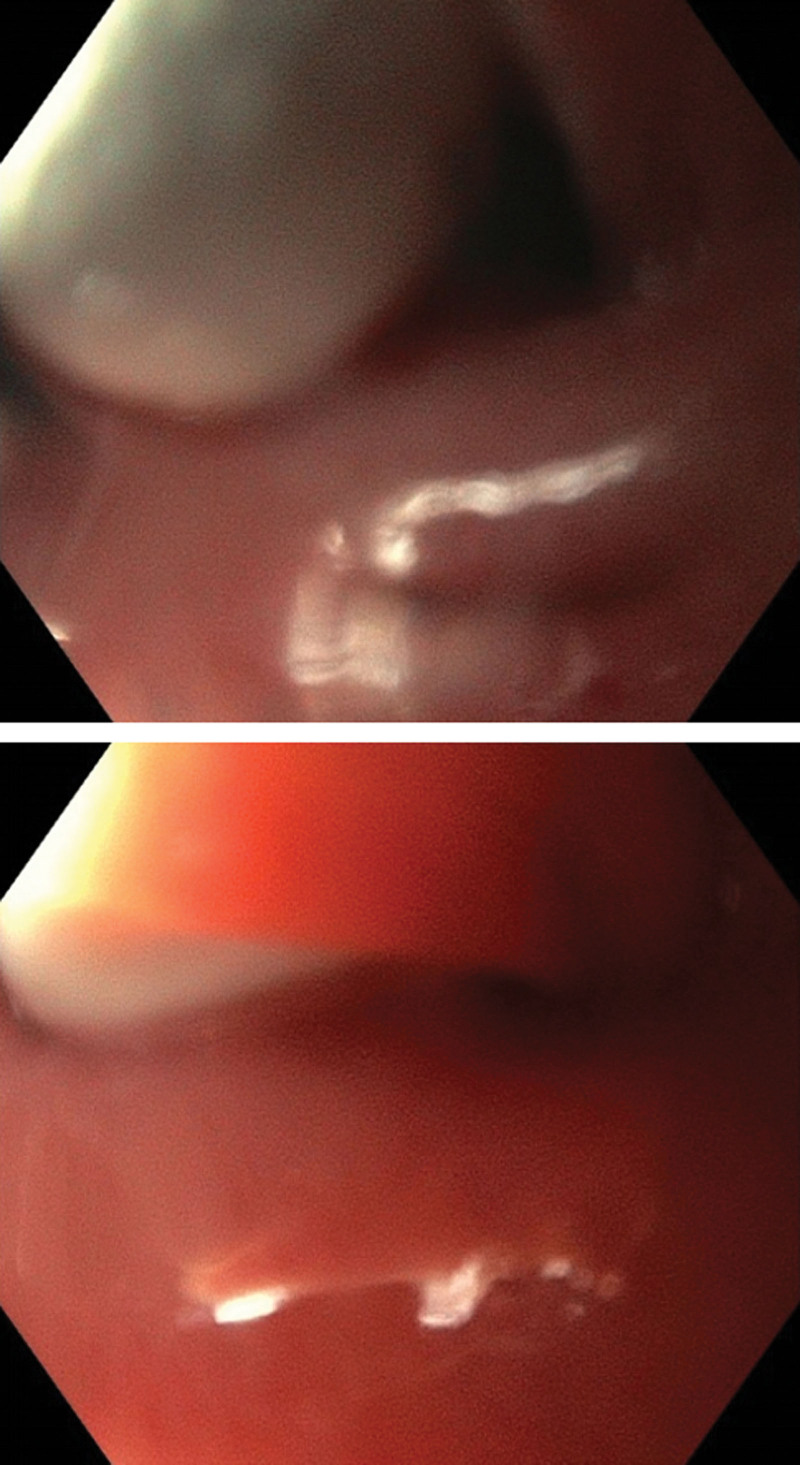
Endoscopic images of capsule endoscopy retention in the pharyngeal pouch.

In the upper esophagus, Zenker’s diverticulum was the most reported area of CR; 84.6%, compared to 15.4% of retentions happening in the cricopharyngeal area; retention in the lower esophagus or other areas were not reported in the literature. The size of Zenker’s diverticulum was reported in 4 patients only, and it ranged between 3 to 9 cm. We noted that retention in most of the incidents happened in medium (2–4 cm) and large (more than 4 cm) size Zenker’s diverticulum, which is expected considering that the size of CE is about 11 mm × 26 mm (Fig. 1).

**Figure 2.**
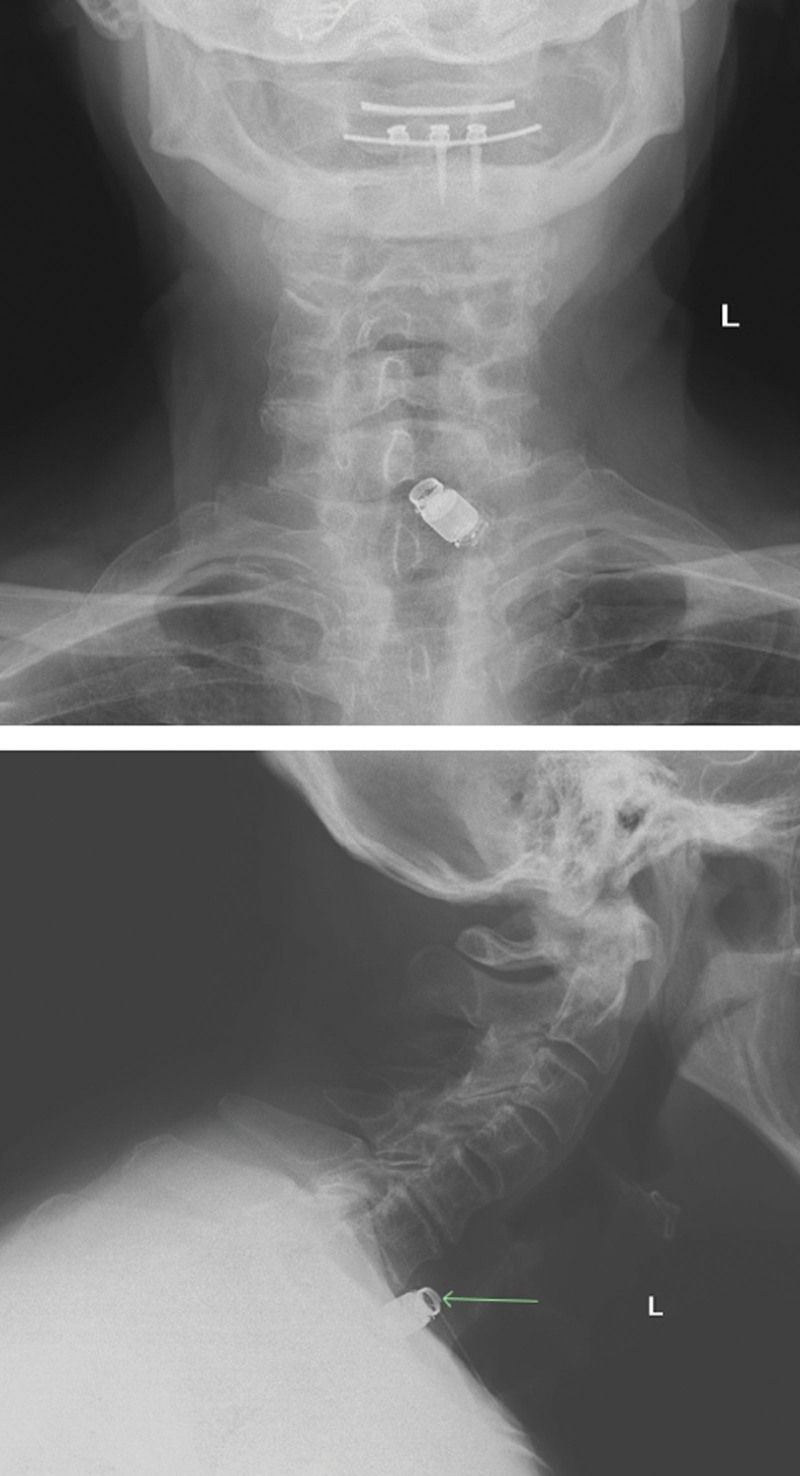
Soft tissue neck x-rays of capsule endoscopy retention in the neck.

Six of the 12 patients were symptomatic due to CR. The main symptoms were discomfort and foreign body sensation in the pharynx, followed by dysphagia; no patients reported respiratory symptoms, bleeding, or other concerning symptoms. CR in the upper esophagus was suspected in three-quarters of patients immediately after ingestion or at the time of assessment of CE images; however, 1 patient presented with dysphagia after 4 months^[22]^ and a second patient presented with dysphagia after 2 years of ingestion.^[24]^ Both patients with delayed symptomatic presentations had plain abdominal radiography showing no foreign body, which was interpreted as CE passage and excretion from the gastrointestinal tract; this points towards the necessity to order neck, chest and abdominal radiography for patients who do not report CE passage 2 weeks after ingestion, especially in elderly patients above the age of 65 years. CE images and plain radiographs of the neck and chest confirmed the diagnosis in all patients (Fig. 2).

**Figure 3.**
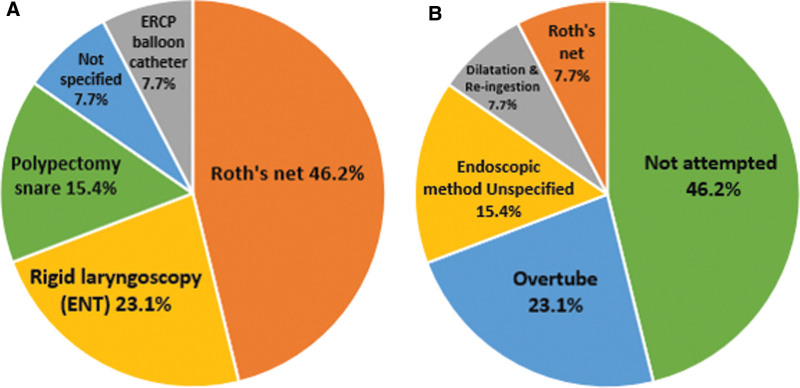
A: Methods of capsule endoscopy retrieval. B: Methods of capsule endoscopy re-insertion. ERCP = Endoscopic Retrograde Cholangio Pancreatography.

Retrieval of the retained CE through conventional endoscopy was successful in most patients (9/12 patients, 76.9%). Roth’s net was used for retrieval in 6 patients, a polypectomy snare in 2 patients, an Endoscopic Retrograde Cholangio Pancreatography balloon catheter in 1 patient and a last patient’s retained capsule was retrieved endoscopically without specifying the tool. In 3 patients (23.1%), the endoscopic retrieval failed despite using the aforementioned tools; these patients had to undergo general anesthetics with retrieval by rigid endoscopy performed by an otolaryngologist with anesthetist support. An essential practical point was the necessity for airway protection during retrieval of CE from the cricopharyngeus, 1 patient had airway intubation, and the second had a balloon catheter inflated at the laryngeal entrance to prevent the capsule aspiration into the airway during retrieval. (Fig. 3A) demonstrates the percentages of each tool used for capsule retrieval.

Endoscopy-guided capsule re-insertion was successful when attempted; most were performed simultaneously with the retrieval procedure. Overtube insertion to bypass the upper esophagus was reported as the safest and most successful method of endoscopic re-insertion; a Savary guidewire and dilator were used in 1 study to insert the overtube,^[16]^ CE was advanced through the overtube using the endoscopy tip, Roth’s net, or polypectomy snare.^[16,17,23]^ One patient had the CE re-inserted using Roth’s net only, and another had the procedure performed endoscopically without specifying the tool used. One patient successfully re-ingested the CE post upper esophageal dilatation a few weeks following CR. Polypectomy snare has been reported to be used for insertion of capsule endoscopy in a patient with Zenker’s diverticulum.^[26]^ (Fig. 3B) demonstrates the percentage of each tool used for endoscopic re-insertion.

All patients with CR had to undergo procedures for retrieval, and a few required admissions monitoring and arranging further tests. Reassuringly, all studies did not report a significant adverse outcome except one that reported signs of inflammation in the Zenker’s diverticulum after CE retrieval; this resolved spontaneously and did not require specific treatment.

In summary, CR in the upper esophagus is uncommon; the exact incidence is difficult to predict due to the low incidence and reporting of this complication. However, it imposes significant morbidity on patients, including exposure to additional anesthetics and endoscopic procedures; it is also associated with the increased cost of testing, endoscopic management, and the potential requirement for a second CE procedure. As far as we know, this is the first study on this topic that included a comprehensive search of databases, followed by an analysis of all published reports for different outcomes and variables. In our review, we were able to summarize the data and provide a general guide of previous experiences of gastroenterologists managing this complication. We noted that CR in the upper esophagus is more prevalent in elderly male patients. Half of the patients had a risk factor for it. Most of the patients were asymptomatic before undergoing the procedure. CR caused a pharyngeal foreign body sensation and dysphagia in half of the patients. Medium-to-large size Zenker’s diverticulum seems to be the most common location for retention; no incidents were reported in smaller diverticula or lower esophagus. Interpretation of CE images and plain soft tissue radiography of the neck are sufficient to diagnose this type of CR. Endoscopic retrieval using Roth’s net was the most reported method; other tools were reported, too; rigid endoscopy with anesthetic support was required when endoscopy failed to retrieve the capsule. Endoscopic re-insertion using an overtube was the safest and most successful method; no failure of re-insertion using endoscopy was reported. A significant adverse outcome related to this issue was not reported.

Our study has a few limitations, expectedly and due to the low incidence of this complication, the number of incidents and patients was small. All the included studies were case reports, and no retrospective or prospective observational studies were found.

## 4. Conclusions

**Figure 4.**
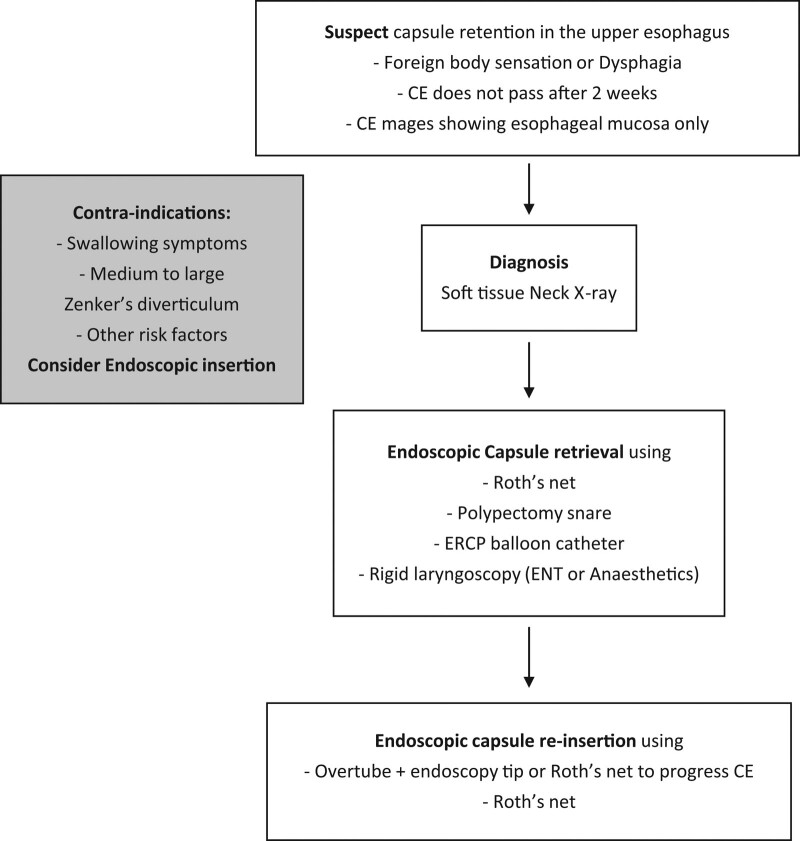
Flow chart of diagnostic and therapeutic tools used in case reports for managing capsule endoscopy retention in the upper esophagus. CE = capsule endoscopy.

We recommend following the current guidelines on considering patients with symptoms of dysfunctional swallowing, including dysphagia, as candidates for endoscopic insertion of CE. Zenker’s diverticulum should be viewed as a relative contra-indication; symptomatic lesions or medium-to-large lesions are likely more appropriate for endoscopic insertion, while the method of insertion of CE in patients with asymptomatic small diverticula remains uncertain; oral ingestion under real-time monitoring can be considered after a discussion with the patient regarding the risk of CR. Patients developing upper esophageal symptoms after CE ingestion should immediately have a plain neck soft tissue and chest radiography. In patients who do not pass the capsule 2 weeks after ingestion, we recommend the addition of plain neck soft tissue and chest radiographs to the currently recommended plain abdominal radiograph, especially in elderly patients above the age of 65 years. Endoscopic retrieval of CE using Roth’s net is probably the first management line; other alternatives include a polypectomy snare or Endoscopic Retrograde Cholangio Pancreatography balloon catheter. If Endoscopic retrieval proves complex or challenging, early otolaryngology with anesthetic consultation is recommended for retrieval using a rigid endoscopy. CE insertion through an overtube to bypass the entrance of the diverticulum seems to be the safest method of endoscopic insertion; CE can be pushed through the overtube using the endoscope tip or Roth’s net. A Savary guidewire followed by the dilator or other tools to guide the overtube insertion should be recommended with a large high-risk diverticulum. (Fig. 4) demonstrates the diagnosis and management of CR in the upper esophagus based on data in the identified case reports.

## Author contributions

**Conceptualization:** Elke Wiseman, Yanna Ko.

**Formal analysis:** Arteen Arzivian, Elke Wiseman.

**Methodology:** Arteen Arzivian.

**Resources:** Arteen Arzivian.

**Software:** Arteen Arzivian.

**Supervision:** Elke Wiseman, Yanna Ko.

**Writing – original draft:** Arteen Arzivian.

**Writing – review & editing:** Elke Wiseman, Yanna Ko.
